# La Crosse Virus Shows Strain-Specific Differences in Pathogenesis

**DOI:** 10.3390/pathogens10040400

**Published:** 2021-03-29

**Authors:** Sarah N. Wilson, Krisangel López, Sheryl Coutermash-Ott, Dawn I. Auguste, Danielle L. Porier, Philip M. Armstrong, Theodore G. Andreadis, Gillian Eastwood, Albert J. Auguste

**Affiliations:** 1 Department of Entomology, College of Agriculture and Life Sciences, Fralin Life Science Institute, Virginia Polytechnic Institute and State University, Blacksburg, VA 24061, USA; swilson3@vt.edu (S.N.W.); klopez5@vt.edu (K.L.); dauguste@vt.edu (D.I.A.); danip@vt.edu (D.L.P.); geastwood@vt.edu (G.E.); 2 Department of Biomedical Sciences and Pathobiology, Virginia Tech, VA-MD College of Veterinary Medicine, Blacksburg, VA 24061, USA; slc2003@vt.edu; 3 Environmental Sciences, Center for Vector Biology and Zoonotic Diseases, The Connecticut Agricultural Experiment Station, New Haven, CT 06504, USA; philip.armstrong@ct.gov (P.M.A.); Theodore.Andreadis@ct.gov (T.G.A.); 4 Center for Emerging, Zoonotic, and Arthropod-Borne Pathogens, Virginia Polytechnic Institute and State University, Blacksburg, VA 24061, USA

**Keywords:** La Crosse virus, encephalitic disease, arbovirus, arbovirus pathogenesis, neurovirulence, neuroinvasiveness

## Abstract

La Crosse virus (LACV) is the leading cause of pediatric viral encephalitis in North America, and is an important public health pathogen. Historically, studies involving LACV pathogenesis have focused on lineage I strains, but no former work has explored the pathogenesis between or within lineages. Given the absence of LACV disease in endemic regions where a robust entomological risk exists, we hypothesize that some LACV strains are attenuated and demonstrate reduced neuroinvasiveness. Herein, we compared four viral strains representing all three lineages to determine differences in neurovirulence or neuroinvasiveness using three murine models. A representative strain from lineage I was shown to be the most lethal, causing >50% mortality in each of the three mouse studies. However, other strains only presented excessive mortality (>50%) within the suckling mouse neurovirulence model. Neurovirulence was comparable among strains, but viruses differed in their neuroinvasive capacities. Our studies also showed that viruses within lineage III vary in pathogenesis with contemporaneous strains, showing reduced neuroinvasiveness compared to an ancestral strain from the same U.S. state (i.e., Connecticut). These findings demonstrate that LACV strains differ markedly in pathogenesis, and that strain selection is important for assessing vaccine and therapeutic efficacies.

## 1. Introduction

The family *Peribunyaviridae* is among the largest known viral families, with >170 known members reported to date. Within this family, the genus *Orthobunyavirus* is a particularly important, and nearly globally distributed, group with an exceptionally diverse vertebrate host and arthropod vector host range [1]. La Crosse virus (LACV) is among the most important members of the California serogroup of orthobunyaviruses and the leading cause of pediatric viral encephalitis in North America [2]. Since the discovery of LACV in 1960 from an infected child presenting with meningoencephalitis in Wisconsin [3], it has quickly emerged as one of the most important arboviral pathogens in North America, with an annual incidence of human disease between 0.004 and 1.6 cases per hundred thousand in the general population of affected areas across the U.S. between 1963 and 2016 [4]; however, this rate is higher in children under 19 years of age (0.09–2.27 cases per 100,000) in these regions [4]. 

LACV virions are pleiomorphic in shape, approximately 100 nm in diameter, and contain an envelope lipid layer that supports the hetero-multimeric glycoproteins [5]. LACV virions encapsidate tripartite negative-sense RNA genomes, including an L (large) segment that encodes a single open reading frame for the RNA-dependent RNA polymerase, an S (small) segment that encodes the nucleoprotein (N) and a small nonstructural protein (NS_S_), and an M (medium) segment which encodes two glycoproteins (G_N_ and G_C_) and a nonstructural protein (NS_M_) [6,7,8]. Previous studies have shown that the NS_S_ of the S segment suppresses the vertebrate type I interferon response, thereby increasing LACV pathogenesis [9]. Other studies have shown that LACV is under polygenic control, but that the M segment plays an important role in its virulence [10,11,12].

LACV-infected persons typically present with a febrile illness lasting up to three days, with nausea, vomiting, and lethargy [13]. However, if the virus invades the central nervous system (CNS), it can result in more severe, and often permanent, CNS deficiencies which are typically observed in children under the age of 16 [2,13,14,15]. In severe cases, LACV infection may result in lifelong neurologic complications and carries an estimated fatality rate of 0.5–1.9% [3,13,16]. The importance of LACV is further exacerbated by the absence of approved interventions such as vaccines or antivirals.

Epidemiological studies show that LACV has historically been predominant in the Midwestern states (Ohio, Wisconsin, Minnesota, Indiana, Illinois, and Iowa), but recently, cases are being reported from northeastern, mid-Atlantic, and southeastern states (North Carolina, Tennessee, West Virginia, Georgia, Virginia, Kentucky, and Rhode Island) of the U.S. [13]. The average number of severe cases has remained relatively constant within the last decade [13]. LACV comprises three, genetically distinct lineages that circulate in different regions of the U.S. [17]. Although an entomological risk exists [18], there is limited evidence of human infection or LACV disease reported in areas where lineage III viruses predominantly circulate within the northeastern U.S., suggesting that this lineage may be attenuated in humans or that there is reduced transmission rates by vectors to humans in these areas [17,18].

LACV has been shown to be both neuroinvasive and neurovirulent in murine models [12,19,20,21,22]. Previous studies showed immune-competent weanling mice to be subject to neurologic disease following intraperitoneal (i.p.) inoculation, but adult mice (greater than six weeks of age) were resistant, showing age-dependent susceptibility. This finding closely mirrors the impact of this virus on humans [12,23]. Other studies have demonstrated that LACV is capable of efficiently replicating within the brain following intracranial (i.c.) inoculation of suckling mice [19,24]. Currently, studies assessing LACV pathogenesis in mice have revolved around lineage I isolates which have shown consistent and comparable pathogenicity (reviewed in [20]); however, no prior studies have employed strains outside of lineage I or compared pathogenesis within the three genetic lineages. Comparing the pathogenesis among LACV strains could provide evidence to explain the epidemiologic differences or disease outcomes observed in humans at risk of infection. Additionally, identifying strains that demonstrate increased virulence is also important to identify challenge viruses for future countermeasure evaluation.

To compare pathogenesis among LACV strains, we evaluated the differences in neuroinvasive potential and neurovirulence among four viral strains from all three genetic lineages in multiple murine models. We also compared ancestral and contemporaneous strains from lineage III from Connecticut to determine potential changes in virulence over time as observed for other arboviruses [25,26]. Our data suggest that LACV strains differ markedly in their pathogenesis, with some strains presenting with reduced neuroinvasive capacity when compared to other strains tested. However, neurovirulence was comparable among strains when administered intracranially. Our studies also show that viruses within lineage III vary in pathogenesis with a recent strain having reduced neuroinvasiveness compared to an ancestral strain from the same state.

## 2. Results

### 2.1. LACV Pathogenesis in Suckling CD-1 Mice 

**Figure 1:**
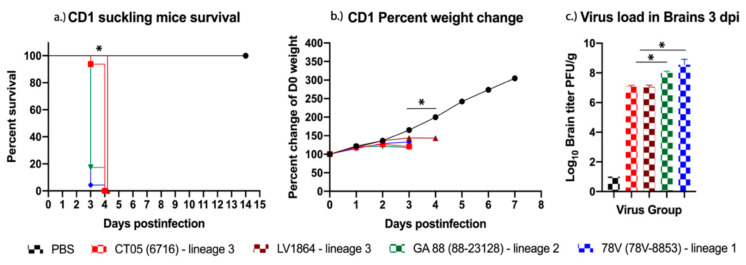
La Crosse virus (LACV) replicates efficiently and causes high mortality rates in suckling mice. In this study, two-day-old suckling mice (*n* = 16–23/group) were challenged intracranially with 10^3^ plaque forming units (PFU) and had (**a**) survival and (**b**) weight change measured daily for 14 days. Only the phosphate buffered saline (PBS) uninfected control mice survived beyond four days postinfection (DPI). (**c**) Virus was detected in brain tissues harvested three DPI, and lineage III strains presented with the lowest titers among the lineages studied. Each data point plotted represents the mean values, and error bars indicate standard deviation. Statistical significance among groups and PBS controls was analyzed by log-rank (Mantel-Cox) test in (**a**) two-way ANOVA with Tukey’s multiple comparison test in (**b**,**c**) Statistical significance is denoted by * (*p* < 0.05).

Representative viruses from all three LACV genetic lineages were rescued and replicated to high titers after a single passage in Vero-76 cells (Table 1). Two strains from lineage III (CT05 and LV1864) and one strain each from lineage I (78V) and II (GA88) were used in this study (Table 1). The pathogenesis of each LACV isolate was evaluated for neurovirulence by i.c. inoculation of two-day-old CD-1 suckling mice. Two litters of mice were intracranially injected with either LACV CT05 (*n* = 16), LV1864 (*n* = 22), GA88 (*n* = 23), 78V (*n* = 23), or a phosphate buffered saline (PBS) negative control (*n* = 12). All mice rapidly gained weight until two days postinfection (DPI), and 100% mortality was observed by four DPI (Figure 1a). Mice infected with CT05 and LV1864 showed comparable weight loss (Figure 1b), but in contrast to 78V and GA88, death was delayed by a single day and showed 100% mortality by four DPI. Weight loss was comparable among viral-infected groups. Brain samples were harvested from a subset of each group (*n* = 3) and used for organ load analysis. All LACV strains replicated efficiently after i.c. inoculation and achieved titers ranging from 6.50 × 10^8^ (in 78V) to 9.40 × 10^6^ (in LV1864) plaque forming units per gram (PFU/g) among infected mice (Figure 1c). Although no significant differences in survival or weight change were observed among viruses, there were significant differences in titers from brain samples taken at three DPI. However, 78V and GA88 demonstrated significantly higher titers than CT05 and LV1864 (*p* < 0.05). 

### 2.2. LACV Pathogenesis in Weanling and Adult Swiss Webster Mice

**Figure 2:**
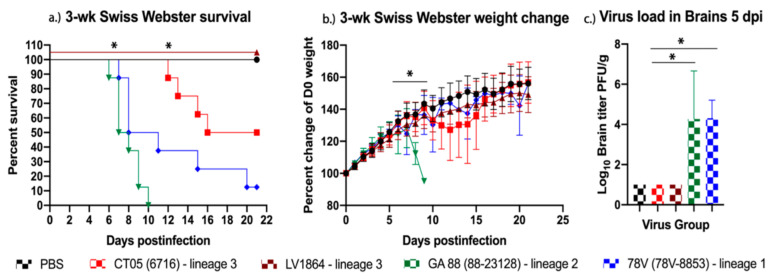
Lineage III La Crosse virus (LACV) strains shows reduced pathogenesis in three-week-old Swiss Webster mice when compared to other lineages. In this study, three-week-old mice (*n* = 14/group) were challenged intraperitoneally with 10^3^ plaque forming units (PFU) of virus (**a**) survival and (**b**) weight change measured daily for 21 days postinfection (DPI). (**c**) Virus was detected in brains harvested five DPI but not three DPI. Lineage III strains presented with reduced neuroinvasiveness and the lowest brain titers among all lineages studied. Each data point plotted represents the mean values, and error bars indicate standard deviation. Statistical significance among groups was analyzed by log-rank (Mantel-Cox) test in (**a**), and two-way ANOVA with Tukey’s multiple comparison test in (**b**,**c**). Statistically significant values are denoted by * (*p* < 0.05).

Next, the pathogenesis of each LACV isolate was evaluated for neuroinvasive capacity by i.p. inoculation of three- and six-week-old Swiss Webster mice. Weanling mice infected with GA88 and 78V virus strains experienced rapid disease presentation, such as weight loss starting at six–seven DPI. Mice later presented with neurological symptoms such as excessive hind limb paralysis, mild seizures, and severe disorientation, after which they were immediately euthanized. The GA88 group succumbed to illness with a 100% mortality rate by 10 DPI. Mice infected with 78V had a slightly reduced rate of mortality but still showed 85% mortality by 20 DPI (Figure 2a). The LACV strain CT05 presented delayed symptoms in mice, not showing weight loss until 10 DPI, although further symptoms were comparable to other strains, and 50% mortality was observed by 16 DPI (Figure 2a). Comparable to PBS controls, mice infected with LV1864 showed no detectable symptoms of disease and maintained healthy weight gain throughout the 21-day study (Figure 2b). No viremia was detected in this weanling mouse model throughout days one–four postinfection, but the virus was detected in a subset of mouse brains harvested at five DPI from the 78V and GA88 virus groups (Figure 2c). Similarly, no significant virus was detected in sera or tissues (liver, spleen, kidney, lung, heart, and testes in males) harvested at three or five DPI. 

**Figure 3:**
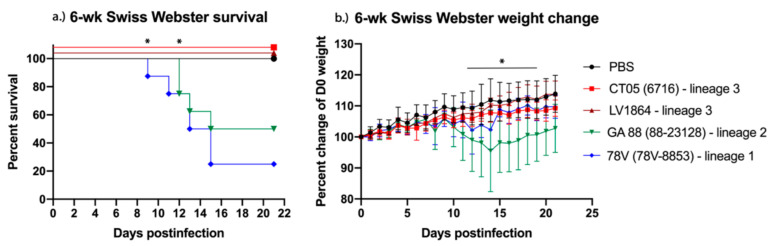
Lineage III La Crosse virus (LACV) strains show reduced pathogenesis in six-week-old Scheme 14. group) were challenged intraperitoneally with 10^3^ plaque forming units (PFU) of virus and (**a**) survival and (**b**) weight change measured daily for 21 days. Lineage III strains presented with no neurological disease in contrast to lineages I and II. Each data point plotted represents the mean values, and error bars indicate standard deviation. Statistical significance among groups was analyzed by log-rank (Mantel-Cox) test in (**a**) and two-way ANOVA with Tukey’s multiple comparison test in (**b**). Statistically significant values are denoted by * (*p* < 0.05).

As expected, the LACV strains that caused the highest morbidity and mortality in weanling mice remained the most pathogenic in the adult mouse model, albeit to a lesser degree. Mice infected with virus strains GA88 or 78V achieved their highest mortality rate by 15 DPI, GA88 with a survival rate of 50%, and 78V with a 25% survival rate (Figure 3a). Mice infected with CT05 or LV1864 showed no signs of disease or mortality throughout this study (Figure 3b). No virus was detected in sera taken at days one–four postinfection or from tissues (liver, spleen, kidney, lung, heart, brain, and testes in males) harvested at either three or five DPI.

## 3. Discussion

A complete understanding of viral pathogenesis, disease burden, and risk of emergence is essential for resource allocation efforts for targeted surveillance, vector control, and the development of therapeutics, including vaccines and antivirals. To evaluate the potential public health significance and the risk and potential burden of disease after infection with various LACV strains, we explored the virulence among four strains from all three LACV lineages circulating within the U.S., using three previously established murine models. Our results provide evidence that LACV strains vary widely in pathogenesis in mice. This is likely a result of reduced neuroinvasiveness which is intrinsic to specific viral strains. Additionally, a contemporaneous lineage III strain from Connecticut showed markedly reduced neuroinvasiveness when compared to an ancestral lineage III strain from Connecticut. 

Results from studies in the weanling and adult mouse models showed that GA88 and 78V remained the most pathogenic and lethal, but clearly presented different timelines for these outcomes. Younger mice were affected at a faster rate than adult mice, showing 50% mortality in the respective strains by day seven (GA88) or eight (78V), compared to days 13 or 15 in adult mice. Mortality rates were also higher in weanling mice. As observed previously, this closely mimics the age-dependency shown in human LACV infections, which is likely a result of a more robust antiviral immune response in adults [20]. These data are also supported by previous studies that showed that lineage I LACV strains demonstrate comparable murine pathogenesis to the strains used herein [11,12,19,20,21,27]. The mechanisms underlying the different manifestation of disease in children compared to adults is not understood and should be explored more fully. 

To evaluate neurovirulence, we utilized a CD-1 suckling mouse model which represents a highly permissive environment for virus replication and is a reliable model for evaluating LACV neurovirulence [19,24]. All LACV strains displayed 100% mortality in suckling mice by four DPI. Each viral strain showed mortality and severe signs of disease requiring euthanasia, including poor mobility, tremors, and lack of weight gain, all strong indicators of encephalitic disease. All virus strains demonstrated comparable neurovirulence, suggesting that neurovirulence was not the reason underlying the reduced pathogenicity observed in peripherally inoculated older mice. Further studies, such as intranasal inoculation of weanling and adult mice, could confirm these findings. Our results also indicated that 78V or GA88 should be preferentially selected for mouse challenge studies, especially when significant disease and lethality are required.

The LACV strains used in our study, excluding the negative control group, showed 0% mortality in the six-week mouse study. Neither the CT05 (lineage III) or LV1864 (lineage III) strain displayed signs of disease throughout the six-week-old mouse model study. This was similarly observed in the three-week mouse model, where mice infected with LV1864 presented little to no signs of disease. The absence of detectable disease in the three-week and six-week models and comparable lethality in the suckling mouse model are strong indicators of a significant difference in the neuroinvasive capacities among strains, as well as within lineage III. Our studies also showed that 78V and GA88 presented with higher titers in brain samples taken from suckling mice and the weanling mouse model, indicating increased fitness for these strains for replication in the brain. However, one limitation of this study is that we were unable to compare viral loads in brain tissues when all infected mouse groups presented with clinical disease. Additional studies are thus needed to assess viral loads present in mice to investigate potential differences in peak viral titer estimates during clinical disease presentation. 

The differential virulence between the LV1864 and CT05 suggested that there may be potential adaptations for reduced murine pathogenesis in comparison to contemporaneous lineage III strains. LV1864 differs from CT05 by only 0.1% nucleotide sequence identity in the S, and 0.2% nucleotide sequence identity in the M segments, whereas partial nucleotide sequences of the L segment (1037 nt) are identical to each other. Additionally, complete M segment comparisons among all four strains showed that CT05 and LV1864 differ from 78V by only 14.5% nucleotide sequence identity (4.5% amino acid sequence identity), and both differ from GA88 by 15.1% nucleotide sequence identity (4.5% amino acid sequence identity). The M segment encodes the antigenic glycoproteins and the NSm protein that is known to attenuate vertebrate type I interferon responses. Given these small sequence differences and the importance of the M segment, further studies should be undertaken to evaluate the genetic differences underlying the altered pathogenesis among the LACV strains in these models. Elucidating the genetic composition or causal mechanisms of this observed attenuation in pathogenesis would benefit rational vaccine design for LACV and other orthobunyaviruses. Previous reverse genetic studies using reassortant LACV strains have shown that LACV pathogenesis may be under polygenic control [10]; thus, it is likely that this difference in pathogenesis may be multigenic in nature.

In summary, our results suggest that there is wide variability in pathogenicity, both between and within LACV lineages. However, further studies comparing more strains from all lineages are needed to confirm these findings. Human seroprevalence studies, specifically, are needed in lineage III endemic regions to identify the extent of exposure to LACV. Nonetheless, lineage III strains demonstrate reduced neuroinvasiveness in comparison to the other strains tested, suggesting that this may be one of the factors influencing the absence of detectable cases and the lower public health significance in endemic regions where this lineage circulates. 

## 4. Materials and Methods

### 4.1. Viruses and Cells Used in the Study

Representative viral strains from three circulating LACV lineages were selected to represent the spatial and temporal distribution of LACV, to include all three genetic lineages, and to have minimal passage histories to reduce tissue cell culture effects on pathogenesis. Viruses were passaged once in Vero-76 (African green monkey kidney cells), purchased from the American Type Culture Collection (ATCC; Manassas, VA, USA) to create stocks for in vivo studies. Briefly, stocks of all strains (see Table 1; LACV 6716-05 (CT05), LV1864, 88-23128 (GA88), and 78V-8853 (78V)) were made by infection of 80% confluent monolayers and incubation at 37 °C for two (for LACV strain 78V-8853 only) or three days (all other strains). Cell culture supernatants were collected and clarified by centrifugation at 4000 rpm for 10 min and stored at −80 °C in 1 mL aliquots. Virus titers were estimated using plaque assays on Vero-76 cells, and are indicated in Table 1. 

### 4.2. LACV Neurovirulence in CD-1 Suckling Mice

The neurovirulence of each virus strain was assessed using a CD-1 suckling mouse model. Ten E17 pregnant dams were obtained from Charles River Laboratories (Wilmington, MA, USA). Mice were allowed to acclimatize and birth pups. Approximately two days after birth, pups were intracranially inoculated with 10^3^ PFU of each virus, and a PBS inoculated group was included as a healthy control. All inoculums were quantified by plaque assays on Vero-76 cells to confirm titers. Suckling mice were weighed and monitored daily for 14 days for signs of disease. On day three postinoculation, brains were harvested from three mice per virus group for virus quantification. Comparing organ titers among strains on day three postinoculation is important for assessing viral fitness through direct comparisons of replication kinetics. Suckling mice were monitored for disease, as described in 4.3 below. Suckling mice were euthanized through manual decapitation because carbon dioxide (CO_2_) euthanasia is ineffective.

### 4.3. LACV Pathogenesis in Adult and Weanling Swiss Webster Mice

Three-week-old weanling and six-week-old adult CFW Swiss Webster mice were obtained from Charles River Laboratories (Wilmington, MA, USA). Mice were separated into six equivalent groups (*n* = 14 for each age group) for infection with CT05, LV1864, GA88, and 78V or with PBS diluent (i.e., healthy controls). Mice were intraperitoneally inoculated with ~10^3^ PFU of the relevant LACV strain diluted in PBS. Mice were weighed and monitored daily for signs of disease for 21 days postinfection [DPI]. Mice were bled via the retro-orbital sinus on one–four DPI (*n* = 7 per group) to assess viremia. On days three and five postinoculation, brains were harvested from three mice per virus group for virus quantification. Tissues were also harvested and used for virus quantification by plaque assay. Comparing organ titers among strains on days three and five postinoculation is important for assessing viral fitness through direct comparisons of replication kinetics and neuroinvasion efficacy. As per the Institutional Animal Care and Use Committee (IACUC) protocols, mice used in this study were monitored daily for any signs of disease. Upon presentation of disease, monitoring increased from 24 h intervals to 6 h intervals. Weights are recorded at 24 h intervals from the time of infection. Disease signals may include lethargy, hunched posture, weight loss, and unresponsiveness. Neurological signs of disease may include seizures, labored breathing, severe disorientation, or paralysis of any kind. Mice were immediately euthanized when presenting neurological signs of disease or upon the loss of 20% or more of their day 0 bodyweight. Mice were euthanized by CO_2_ asphyxiation until breathing was absent, before being bled by cardiac puncture. Death was confirmed using a mechanical method such as cervical dislocation. 

### 4.4. Statistical Analysis

Data normalcy was tested using a combination of Q-Q plot and box plot analyses. Two-way ANOVAs with a Tukey’s multiple comparison test were used to analyze weight and viremia data, and a log-rank Mantel-Cox test was used to analyze the Kaplan-Meier survival curves using GraphPad Prism software (version 8).

### 4.5. Ethical Approval 

All experimental protocols were approved by the Virginia Tech Institutional Biosafety Committee under protocol number 17-052 on 5 April, 2018. All procedures involving animals were approved on 8 October, 2018 by IACUC at Virginia Tech, and all animal experiments were performed in compliance with the guidelines of the IACUC at Virginia Tech.

## Figures and Tables

**Table 1:** Associated metadata for each La Crosse virus (LACV) strain used in the study.

Strain Designation	Viral Strain	Year of Isolation	Location	Lineage	Titer (PFU/mL)
* CT05	6716	2005	Fairfield, CT	III	2.73 × 10^6^
LV1864	LV1864	2018	Redding, CT	III	1.07 × 10^7^
* GA88	88-23128	1988	Tifton, Ga	II	5.22 × 10^6^
* 78V	78V-8853	1978	Rochester, MN	I	6.29 × 10^7^

* Viruses provided by Armstrong and Andreadis.
